# Functional Diversity of Genes for the Biosynthesis of Paeoniflorin and Its Derivatives in *Paeonia*

**DOI:** 10.3390/ijms140918502

**Published:** 2013-09-09

**Authors:** Yuan Yuan, Jun Yu, Chao Jiang, Minhui Li, Shufang Lin, Xumin Wang, Luqi Huang

**Affiliations:** 1 National Resource Center for Chinese Materia Medica, Academy of Chinese Medical Sciences, Beijing 100700, China; E-Mails: yuanyuan@icmm.ac.cn (Y.Y.); jiangchao0411@126.com (C.J.); li_minhui@aliyun.com (M.L.); linshufang2006@126.com (S.L.); 2 CAS Key Laboratory of Genome Sciences and Information, Beijing 100029, China; E-Mail: junyu@big.ac.cn

**Keywords:** *Paeonia*, bark, orthologs, gene expression, paeoniflorin, and gallic acid

## Abstract

The *Paeonia* root, with or without bark, are considered vital traditional Chinese medicine materials; the examples are those of *Bai Shao*, *Chi Shao*, and *Dan Pi.* In this study, we examine 24 genes and their expressions involved in the biosynthesis of paeoniflorin and its derivatives, which are active compounds of the *Paeonia* root, in *Paeonia lactiflora* and *P. suffruticosa*, as well as other related plants, *Punica granatum*, *Rhus radicans*, and *Coriaria nepalensis*. Our phylogenetic analyses suggest that these genes have functional diversity, and analysis of the transcriptional level shows paeoniflorin and gallic acid biosynthesis-related genes exhibit different transcription profiles in flowers, carpels, bark-free roots, and bark of *P. lactiflora*. The correlation analysis of gene expression and active compound contents support the idea that hydroxymethylglutaryl-CoA synthase and phosphomevalonate kinase in the mevalonate pathway and 3-dehydroquinate dehydratase/shikimate dehydrogenase in shikimate biosynthesis are potentially closely related to the accumulation of paeoniflorin and benzoylpaeoniflorin. Coupling gene diversity with chemical analysis, we show that paeoniflorin and its derived aromatic amino acids are predominant in bark.

## 1. Introduction

In China, Korea, and Japan, a decoction of the dried root of *Shao Yao* (*Paeonia lactiflora* Pall.) is used to treat rheumatoid arthritis, systemic lupus erythematosus, hepatitis, dysmenorrhea, muscle cramps and spasms, and fever for more than 1200 years [1]. In modern herb markets, the dry bark-free root of cultivated *P. lactiflora* is called *Bai Shao* (literally, “White Peony”) and the dry root with bark of the wild *P. lactiflora* is called *Chi Shao* (literally, “Red Peony”). However, consensus on their characteristics, including flower color, region of cultivation, medicinal processing, and origin of domestication, has yet to be reached [2]. *Dan Pi* (literally, “Tree Peony Bark”), another traditional Chinese medicine material, is derived from the root cortex of *P. suffruticosa* and serves as a remedy for various diseases, including macula, epilepsy, and menstrual disorders [3].

Differences between *Bai Shao*, *Chi Shao*, and *Dan Pi* can be attributed to the functional difference of the barks. A water/ethanol extract of *Bai Shao* has been found to contain all glucosides of peony, which include paeoniflorin, albiflorin, oxypaeoniflorin, benzoylpaeoniflorin, oxybenzoylpaeoniflorin, paeoniflorigenone, lactiflorin, galloylpaeoniflorin, paeonin, paeonolide, and paeonol [4,5], most of which are monoterpeneglucosides. Paeoniflorin is most abundant (>90%) among them and accounts for the pharmacological effects observed for the total extract of peony glucosides in both *in vitro* and *in vivo* studies. *Chi Shao* shares similar chemical compounds with *Bai Shao* but also shows subtle differences from it [6]. *Dan Pi* has been reported to exhibit antiproliferative, anti-inflammation, anti-infection, antihypertensive, antidiabetic, and neuroprotective activities [7]. The active compounds of *P. suffruticosa*, such as paeonol and paeoniflorin, have also been previously identified [8,9]. Although all these three traditional Chinese medicine materials from Paeonia plants have paeoniflorin and gallic acid, as well as their derivatives, their different anatomic parts and the compound distributions contribute directly to their distinct pharmacological effects.

**Figure 1:**
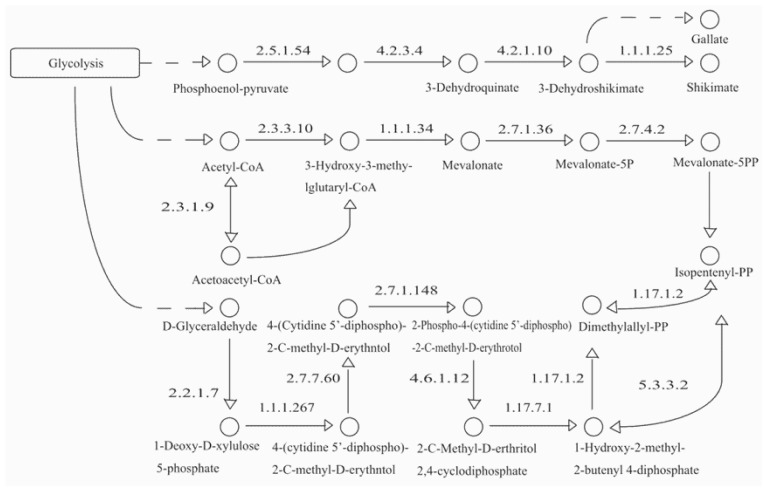
The biosynthesis of Isopentenyl-PP and gallate.

A commercial preparation of the total peony glucoside extract was approved by the State Food and Drug Administration of China as a disease-modifying drug for rheumatoid arthritis in 1998. From its chemical structure, we speculate that benzoylpaeoniflorin and galloylpaeoniflorin originate from paeoniflorin with benzoic acid and gallic acid, respectively (Figure S1). Paeoniflorin is a monoterpene glucoside in which the biosynthesis is controlled by the terpene pathways, including the mevalonate (MVA) and 1-deoxy-d-xylulose-5-phosphate/methyl-erythritol-4-phosphate DXP/MEP pathways (Figure 1). Both the MVA and DXP/MEP pathways lead to the synthesis of the universal precursor, isopentenyl-PP. In the MVA pathway, the key biosynthesis enzymes include hydroxymethylglutaryl-CoA synthase (HMGCS; 2.3.3.10), acetyl-CoA *C*-acetyltransferase (atoB; 2.3.1.9), HMG-CoA reductase (NADPH) (HMGCR; 1.1.1.34), mevalonate kinase (MVK; 2.7.1.36), and phosphomevalonate kinase (PMK; 2.7.4.2). The key biosynthesis enzymes in the DXP/MEP pathway include 1-deoxy-d-xylulose-5-phosphate synthase (DXPS; 2.2.1.7), 1-deoxy-d-xylulose-5-phosphate reductoisomerase (DXR; 1.1.1.267), 2-*C*-methyl-d-erythritol 4-phosphate cytidylyltransferase (ispD; 2.7.7.60), 4-diphosphocytidyl-2-*C*-methyl-d-erythritol kinase (ispE; 2.7.1.148), 2-*C*-methyl-d-erythritol 2,4-cyclodiphosphate synthase (ispF; 4.6.1.12), (E)-4-hydroxy-3-methylbut-2-enyl-diphosphate synthase (HDS; 1.17.7.1), 4-hydroxy-3-methylbut-2-enyl-diphosphate reductase (HDR; 1.17.1.2), and isopentenyl-diphosphate delta-isomerase (IDI; 5.3.3.2). Benzoic acid and gallic acid are also important compounds in aminobenzoate degradation (KO00627) under the catalytic activities of 3-deoxy-7-phosphoheptulonate synthase (aroA; 2.5.1.54), 3-dehydroquinate synthase (aroB; 4.2.3.4), and 3-dehydroquinate dehydratase/shikimate dehydrogenase (aroDE; 4.2.1.10/1.1.1.25).

A number of studies have reported the functional genes in *P. lactiflora and P. suffruticosa*. For instance, a mitochondrial phosphate transporter was isolated from a subtractive cDNA library from the bursting buds of *P. suffruticosa* and its expression in response to chilling treatment during the breakage of bud dormancy [10]. Despite the fact that the expression pattern of flavonoid biosynthetic genes in the flower of herbaceous peony was investigated [11], data on genes related to the active compounds of *Paeonia* remain elusive.

In this paper, we obtained genes from the biosynthetic pathway of paeoniflorin and gallic acid in the *P. lactiflora* transcriptome. We also selected the transcriptome databases of three other plants, for comparative purposes, namely, *Punica granatum*, *Rhus radicans*, and *Coriaria nepalensis*, which contain gallic acid but not paeoniflorin (Table S1) [12–16]. To investigate specific genes related to the biosynthesis of paeoniflorin and gallic acid in *P. lactiflora* as evidence to distinguish *Bai Shao* and *Chi Shao*, we not only analyzed phylogenetic relationships of the relevant genes from *P. lactiflora*, *P. suffruticosa*, *P. granatum*, *R. radicans*, and *C. nepalensis*, but also their expressions, and contents of the active compounds in different anatomic parts of *P. lactiflora.*

## 2. Results

### 2.1. Identification of 24 Paeoniflorin and Gallic Acid Biosynthesis-Related Genes in *P. lactiflora* and Other Related Plants

Using a systematic computational approach, we identified 24 novel paeoniflorin and gallic acid biosynthesis-related genes from the current *P. lactiflora* transcriptome assembly (Table 1). These genes are members of a group of 17 genes that encode all the enzymes involved in the biosynthesis of paeoniflorin and gallic acid (Figure 1). Only eight genes have full-length cDNAs, and their deduced amino acids showed different lengths, isoelectric points, molecular weights, subcellular localizations, and signal peptides. These genes share highly conserved domains and motifs with the genes in their gene family, respectively (Table 2).

The genes were identified based on sequence homology to known sequences and domains. For instance, PLDHS1 protein was recognized by its conserved domain of DAHP synthetase (class II, IPR002480). PLHMGCSs protein has the conserved active site (IPR000590) of Hydroxymethylglutaryl-coenzyme A synthase. PLACAT and PLDXP contain their conserved domains of Thiolase (IPR002155) Deoxyxylulose-5-phosphate synthase (IPR005477), respectively. We also identified many orthologous genes in other related plants, such as 28 in *P. suffruticosa*, 23 in *P. granatum*, 28 in *R. radicans*, and 38 in *C. nepalensis*, based on Blast comparison and annotations referred to KEGG and Interpro (Table S5). Not all orthologous genes were found; for instance, E2.7.7.60 was absent in the data from *P. granatum*.

### 2.2. Phylogenetic Analysis of Paeoniflorin and Gallic Acid Biosynthesis-Related Orthologs

**Figure 2:**
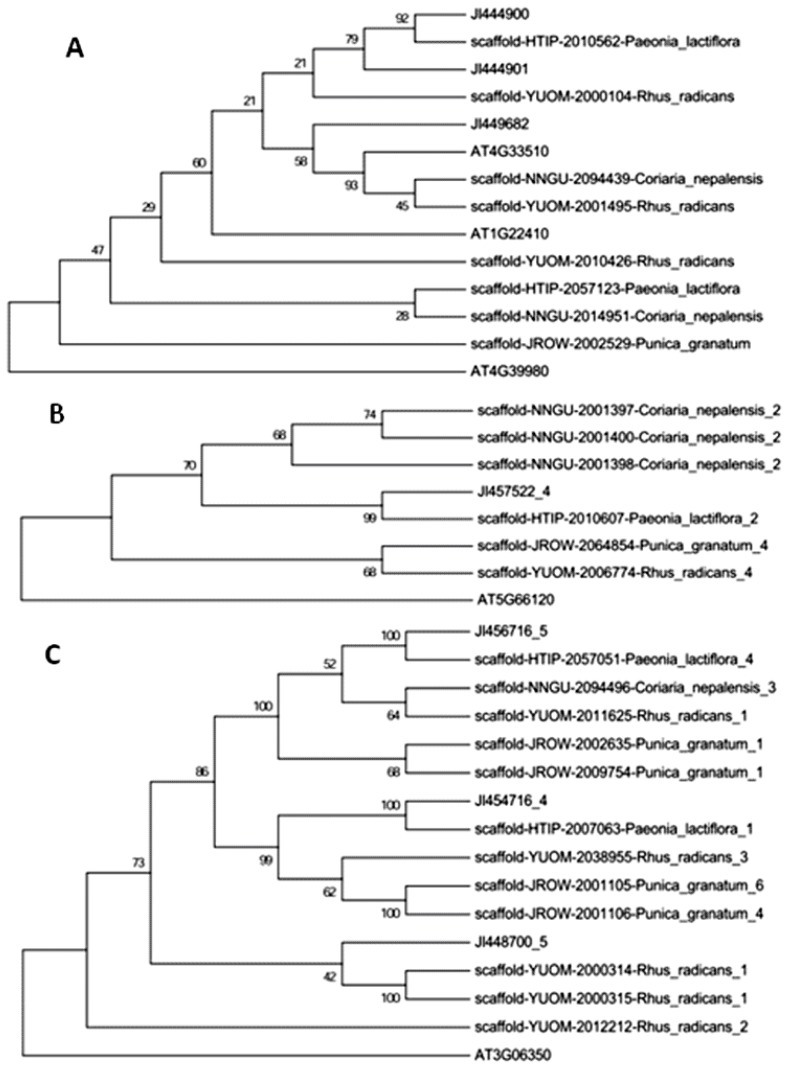
Phylogenetic relationship of gallic acid biosynthesis-related genes. (**A**) DHS, E2.5.1.54; (**B**) DHQS, E4.2.3.4 and (**C**) aroDE, E4.2.1.10/1.1.1.25. The rooted neighbor-joining tree was constructed with ClustalW [18]. HTIP, *P. lactiflora*; JI, *P. suffruticosa*; JROW, *P. granatum*; YUOM, *R. radicans*; NNGU, *C. nepalensis*; and AT, *A. thaliana*.

**Figure 3:**
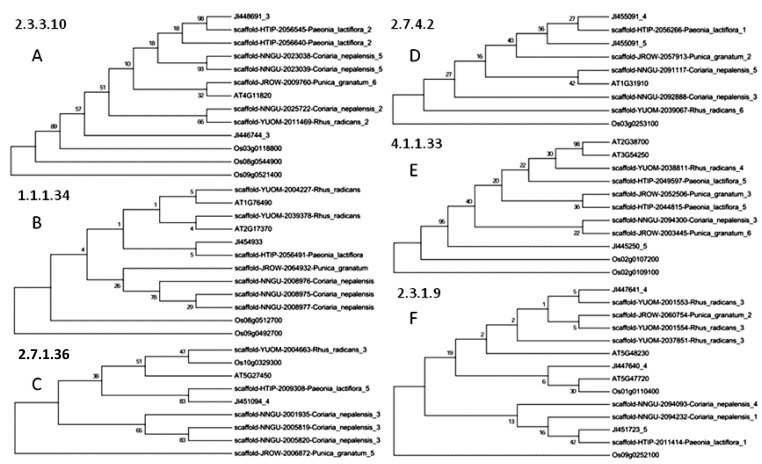
Phylogenetic relationship of paeoniflorin biosynthesis-related genes. (**A**) HMGS, E2.3.3.10; (**B**) HMGCR, E1.1.1.34; (**C**) MVK, E2.7.1.36; (**D**) PMK, E2.7.4.2; (**E**) MVD, E4.1.1.33; and (**F**) ACAT, E2.3.1.9. The rooted neighbor-joining tree was constructed with ClustalW [18]. HTIP, *P. lactiflora*; JI, *P. suffruticosa*; JROW, *P. granatum*; YUOM, *R. radicans*; NNGU, *C. nepalensis*; and AT, *A. thaliana*.

Phylogenetic analysis shows that the genes we identified are those involved in the biosynthesis of terpenes and aminobenzoate degradation in *Arabidopsis thaliana* and *Oryza sativa*, and branches with bootstrap values above 50% were analyzed (Figures 2 and 3; Figures S2 and S3). We also observe significant sequence divergence, as it is related to genetic distance and functional relevance [17]. One example is shown in Figure 2A, where *PLDHS* HTIP-2010562 and HTIP-2057123 (E2.5.1.54) are functionally diversified as their sequences change. Similarly, *PLaroDE* HTIP-2007063 and HTIP-2057051 (E4.2.1.10/1.1.1.25) may also be functionally differentiated (Figure 2C).

A pair of orthologous genes in *P. lactiflora* (*PLDXP3*, HTIP-2057315) and *P. suffruticosa (*JI447843) may also be evolved for differentiated functions as compared with the other genes (Figure S2). There are many orthologous genes among *P. suffruticosa*, *P. granatum*, *R. radicans*, and *C. nepalensis*, which exhibit conserved homology and may share their functions (Figure S3).

### 2.3. Tissue-Specific Accumulation of Active Compounds in *P. lactiflora*

**Figure 4:**
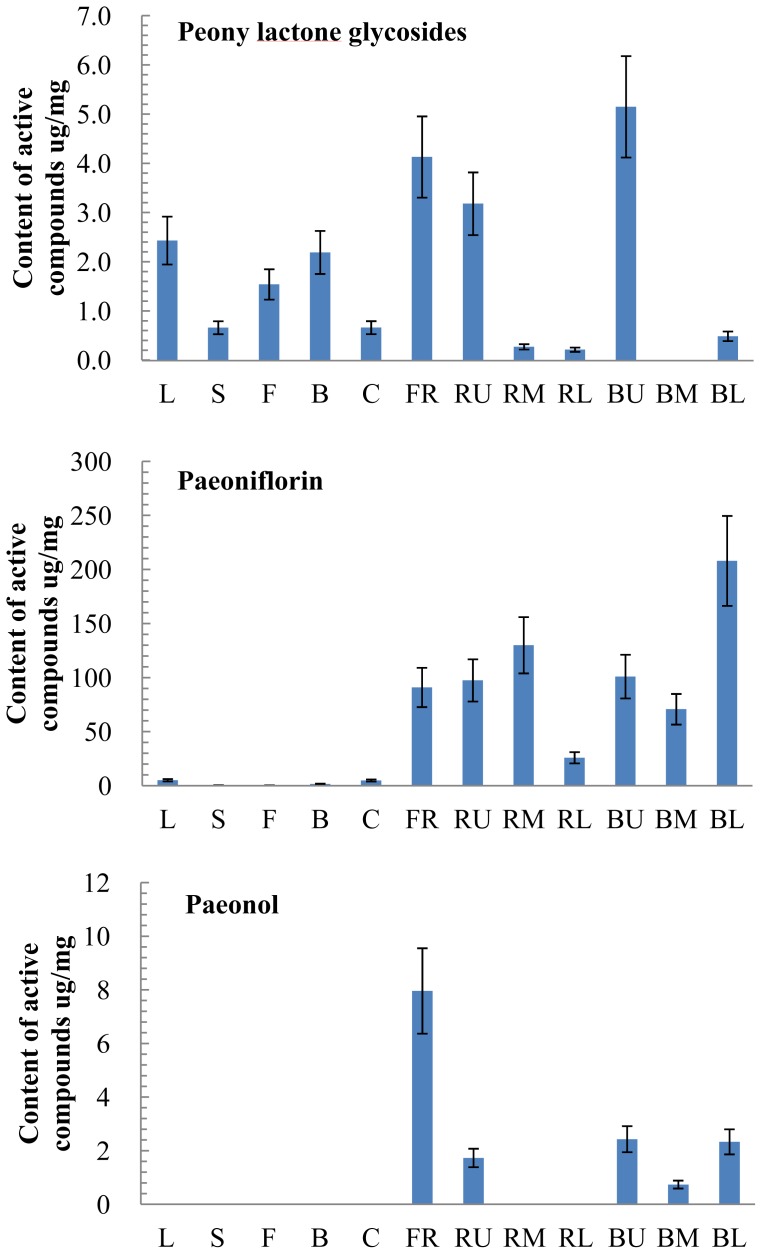

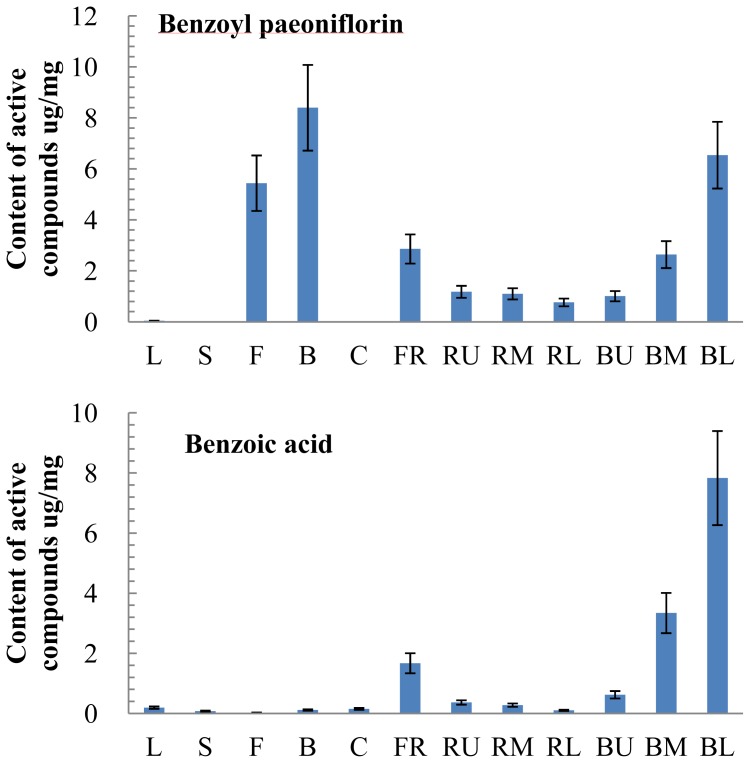
The active compound contents in different tissues of *P. lactiflora*. L, leaf; S, stem; F, flower; B, bud; C, carpel; FR, fibrous root; RU, RM and RL, the upper, middle, and lower parts of the bark-free root; BU, BM, and BL, the upper, middle, and lower portions of the root bark.

To study the tissue-specific accumulation of the shared active compounds of medicinal plant group *Paeonia*, which include peony lactone glycosides, paeoniflorin, paeonol, benzoylpaeoniflorin, and benzoic acid, in both *P. lactiflora* and *P. suffruticosa*, we first determined the contents of these compounds in various plant tissues (leaves, stems, flowers, buds, carpels, fibrous roots, and roots with or without bark) of *P. lactiflora* (Figure 4). Peony lactone glycosides are the major compounds in leaves, stems, and carpels, but present in almost all tissues in variable contents. Both peony lactone glycosides and benzoylpaeoniflorin are the major compounds in flowers and buds.

As roots are the medicinal organs here, we divided roots into different portions for the analysis. The contents of peony lactone glycosides are higher in fibrous roots and upper roots than in the middle and lower roots and the fact suggests that the content of peony lactone glycosides is growth related. In addition, the contents of paeoniflorin and benzoylpaeoniflorin appeared bark-associated in that their contents are higher in the bark of lower roots than in the lower bark-free roots. Similarly, the contents of paeonol and benzoic acid are higher in the bark of middle and lower roots than in the bark-free middle and lower roots. Furthermore, our correlation analysis showed that the content of benzoic acid is closely related to that of benzoylpaeoniflorin (*R* = 0.99; Table S6). These results suggest that the accumulation of most compounds coincides with root growth and some are bark-associated.

### 2.4. Expression of Gallic Acid Biosynthesis-Related Genes in Different Tissues of *P. lactiflora*

**Figure 5:**
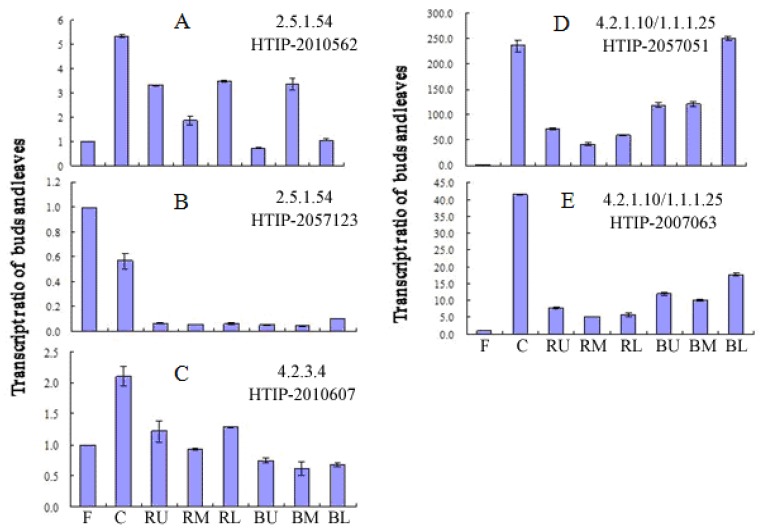
The transcription level of gallic acid biosynthesis-related genes in different tissues of *P.lactiflora*. (**A**,**B**) DHS, E2.5.1.54; (**C**) E4.2.3.4; and (**D**,**E**) aroDE, E4.2.1.10/1.1.1.25 in different tissues of *P. lactiflora*. F, flower; C, carpel; RU, RM and RL, the upper, middle, and lower parts of the bark-free root; BU, BM and BL, the upper, middle, and lower portions of the root bark.

Among the gallic acid biosynthesis-related genes, HTIP-2010562 and HTIP-2057123 (*PLDHS*, E2.5.1.54) exhibit different transcription profiles in flowers, carpels, bark-free roots, and bark. *PLDHS1* (HTIP-2010562) has a higher transcription level than *PLDHS2* (HTIP-2057123) in roots and bark, where active compounds accumulate, and this result suggests the functional importance of *PLDHS1* in the synthesis of gallic acid. In addition, HTIP-2007063 and HTIP-2057051 (*PLaroDE*, E4.2.1.10/1.1.1.25) exhibit similar transcription profiles in different tissues. The transcription levels of *PLaroDE1* and *PLaroDE2* are higher in bark than in bark-free roots. The change in *PLDHQS (*HTIP-2010607) was only found in bark-free roots, bark of the lower roots and carpel (Figure 5).

### 2.5. Expression of Paeoniflorin Biosynthesis-Related Genes in Different Tissues of *P. lactiflora*

**Figure 6:**
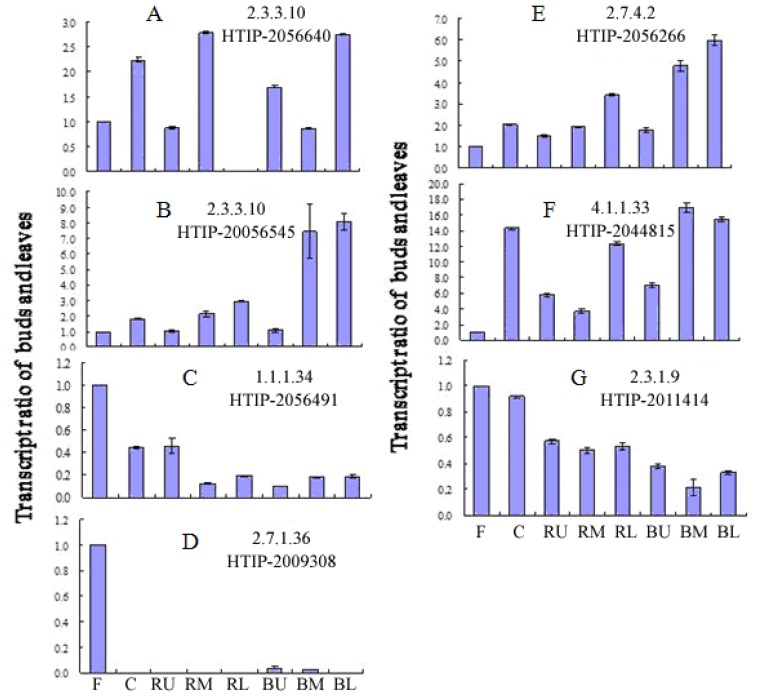
The transcription level of paeoniflorin biosynthesis-related genes in different tissues of *P. lactiflora*. (**A**,**B**) HMGS, E2.3.3.10; (**C**) HMGCR, E1.1.1.34; (**D**) MVK, E2.7.1.36; (**E**) PMK, E2.7.4.2; (**F**) MVD, E4.1.1.33; and (**G**) ACAT, E2.3.1.9 in different tissues of *P. lactiflora*. F, flower; C, carpel; RU, RM and RL, the upper, middle, and lower portions of the bark-free root; BU, BM, and BL, the upper, middle, and lower portions of the root bark.

In the MVA pathway, the transcription levels of *PLHMGCR* (HTIP-20156491) and *PLACAT* are lower in roots than in flowers. However, the transcription level of *PLACAT* is higher in the bark-free root than in the bark. In addition, *PLHMGS2* (HTIP-2056640), *PLPMK* (HTIP-2056266), and *PLMVD1* (HTIP-2044815) exhibit similar transcription profiles that are higher in roots than in flowers (Figure 6). In the MEP/DXP pathway, *PLDXR2* (HTIP-2006951), *PLIspF* (HTIP-2054028), *PLHDS* (HTIP-2007108), *PLIspD* (HTIP-2008416), *PLHDR* (HTIP-2056592), *PLCMK (*HTIP-2008205), and *PLIDI* (HTIP-2055407) display similar transcription profiles among all tissues (Figure S4). Some differential expressions were found for the three *PLDXPS*s, HTIP-2003675, HTIP-2057315, and HTIP-203301 (Figure S5).

### 2.6. Correlation of Gene Expression and Active Compound Contents

Among the five gallic acid biosynthesis-related genes of shikimate biosynthesis, only HTIP-2057051 and HTIP-2007063 encoding aroDE (E4.2.1.10/1.1.1.25) are closely related to the contents of benzoic acid and benzoylpaeoniflorin, with correlation coefficients higher than 0.8 (Table 3). In the MVA pathway, the transcription levels of *PLHMGS1* (HTIP-2054545) and *PLPMK (*HTIP-2056266) were also deemed closely related to the contents of benzoic acid and benzoylpaeoniflorin, whereas HTIP-2056640 was closely related to the content of paeoniflorin (*R* = 0.89).

## 3. Disscussion

### 3.1. Preferential Expression of Gene Families in Bark

Historically, the function of *P. lactiflora* bark has always been a matter of debate. The analysis of the chemistry and genomics of *Chi Shao* and *Bai Shao* can help in defining it. Chemical analysis showed that the contents of paeoniflorin and benzoylpaeoniflorin are higher in the lower parts of the bark. The contents of paeoniflorin in the upper and middle parts of the bark were not significantly different from that in roots without bark, which can be attributed to the weight ratio of bark to whole roots. The transcription levels of *PLaroDE*s (HTIP-2057051 and HTIP-2007063) in shikimate biosynthesis as well as *PLMVK* (HTIP-2009308), *PLPMK* (HTIP-2056266), and *PLMVD*1 (HTIP-2044815) in the mevalonate pathway were higher in bark than in roots without bark. The results of the transcription profiles showed that paeoniflorin and its derivative aromatic amino acid biosynthesis-related genes have higher expressions in bark, suggesting their higher contents therein. Our results from gene expression and chemical analysis showed that paeoniflorin and its aromatic amino acid derivatives were predominant in bark, thus supporting the view that bark is the discriminating factor. In addition, the transcription levels of PLACAT (HTIP-2011414) and PLDXPS1 (HTIP-203301) were higher in roots without bark than in bark, suggesting that other compounds may also have important functions in the pharmacological activity of *Bai Shao*.

### 3.2. Variations of Key Genes in Shikimate Biosynthesis and the MVA Pathway Lead to Active Compound Accumulation

Paeoniflorin and its aromatic amino acid derivatives, such as benzoylpaeoniflorin, are medicinally active compounds in *P. lactiflora*. In addition, the biosynthesis of paeoniflorin and benzoylpaeoniflorin is branched from glycolysis and involved shikimate and terpenoid biosynthesis. To investigate which genes govern the accumulation of paeoniflorin and its derivatives in *P. lactiflora*, we analyzed the root-associated expression of key genes in shikimate and terpenoidbiosyntheses, couple with the correlation of their transcriptions and active compound contents. In plants, the shikimate pathway leads to the biosynthesis of aromatic amino acids, and the bifunctional aroDE catalyzes the conversion of dehydroquinate into shikimate. Suppressed aroDE expression in tobacco reduces the content of aromatic amino acids and downstream products [19]. In the root of *P. lactiflora*, HTIP-2057051 and HTIP-2007063 of the aroDE gene family (E4.2.1.10/1.1.1.25) in shikimate biosynthesis are closely related to the accumulation of benzoic acid and benzoylpaeoniflorin. Paeoniflorin is a monoterpene glucoside produced via the mevalonate-independent pathway, as with other monoterpenes [20]. HMG-CoA synthaseof Arabidopsis is an enzyme of the mevalonate pathway involved in the biosynthesis of isoprenoids, such as sterols [21]. An improvement in HMG-CoA synthase has been reported to increase MVA production in *Escherichia coli* [22]. PMK was found to catalyze a key step in isoprenoid/sterol biosynthesis, converting mevalonate 5-phosphate and ATP to mevalonate 5-diphosphate and ADP [23]. Expression analysis revealed the importance of HMG-CoA synthase, MVK, and other genes in the provision of precursors for rubber biosynthesis [24]. Overexpression of HMGS reportedly up-regulates HMGR, leading to enhanced sterol content and stress tolerance in Arabidopsis [25]. Our results also showed that HTIP-2054545 and HTIP-2056640 (the HMG-CoA synthase gene family, E2.3.3.10) and HTIP-2056266 (the *PMK* gene family, E2.7.4.2) in the mevalonate pathway are potentially closely related to the accumulation of paeoniflorin and benzoylpaeoniflorin. The content of paeoniflorin was used as a standard to evaluate the chemical quality of *P. lactiflora* in *The Chinese Pharmacopoeia*, and increasing the expression of the above-mentioned genes may improve the content of paeoniflorin in the roots and the chemical quality of *P. lactiflora*.

### 3.3. Gene and Functional Diversity of Paeoniflorin and Gallic Acid Biosyntheses Pathway

The functional divergence of enzymes is often a result of gene duplication followed by sequence variation, known as neofunctionalization and subfunctionalization [26,27]. Paeoniflorin is unique to *Paeonia* but aromatic amino acids, such as gallic acid, are abundant in most of plants, e.g., *P. lactiflora*, *P. suffruticosa*, *P. granatum*, *R. radicans*, and *C. nepalensis*. Based on phylogenetic analysis, 14 paralogs and orthologs were used to predict the functional diversity of the active compounds. In gallic acid biosynthesis, PLaroDE paralogs could have the same function, and their orthologs were also found in *P. suffruticosa*, *P. granatum*, *R. radicans*, and *C. nepalensis*. According to the results of gene expression analysis, aroDE orthologs and paralogs had a similarly expression pattern in different tissues and are related to the contents of the aromatic amino acids. In paeoniflorin biosynthesis, PLDXPS paralogs may represent a distinct function based on phylogenetic analysis and showed diverse expressed patterns in different tissues. These findings suggest that the complexity of orthologs and paralogs in paeoniflorin and gallic acid biosynthesis can lead to differences in their functional diversity and chemical compound contents.

## 4. Experimental Section

### 4.1. Plant Materials

**Figure 7:**
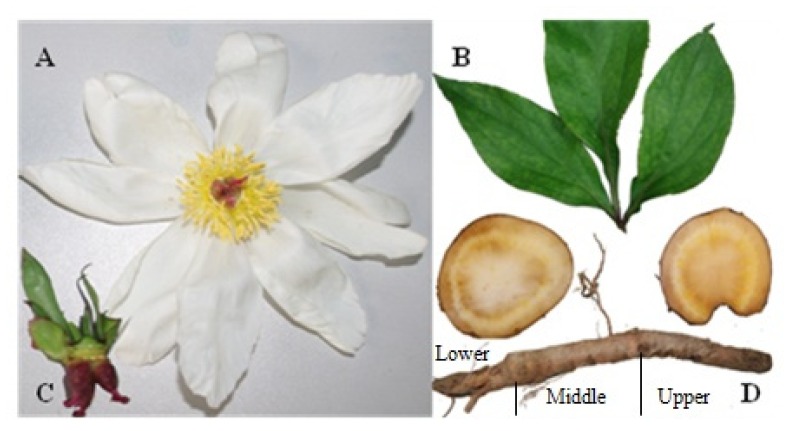
Plant materials of *P. lactiflora*. (**A**) flower; (**B**) leaf; (**C**) carpel; and (**D**) root.

Fresh leaves, flowers, carpels, and roots of *P. lactiflora* were collected from the Medicinal Plant Garden of Beijing University of Chinese Medicine (Figure 7). The root trunk was divided into three parts, the upper, the middle, and the lower. Each root part was further divided into root without bark and bark only. Five plants were sampled and authenticated by Chunsheng Liu (Beijing University of Chinese Medicine, Beijing, China).

### 4.2. Gene Prediction and KEGG Annotation

The current transcriptomic assemblies of *P. lactiflora*, *P. granatum*, *R. radicans*, and *C. nepalensis* were from the 1 kP Project (http://www.1kp-project.com) and the cDNA assemblies of *P. suffruticosa* were retrieved from the public database (GenBank; Available online: http://www.ncbi.nlm.nih.gov). The sequences were annotated based on KEGG [28] and orthologs of the paeoniflorin and gallic acid biosynthesis-related genes from various plant species were identified by using BLASTx [29] with an *e*-value cutoff of 10^−5^. All retrieved sequences from the Genscan web server (http://genes.mit.edu/GENSCAN.html) were used for gene prediction [30]. The predicted gene models were further examined and corrected manually by comparison with related genes identified from other plant species.

### 4.3. Sequence and Phylogenic Analyses

Theoretical isoelectric points and molecular weights were predicted using the Compute pI/MW tool on the ExPASy server (http://web.expasy.org/compute_pi/) [31]. The localizations of the deduced proteins were predicted on the TargetP 1.1 server (http://www.cbs.dtu.dk/services/TargetP/) as well as iPSORT and PSORT programs (http://ipsort.hgc.jp/). Conserved domains were searched against the Pfam protein family database with InterProScan (http://www.ebi.ac.uk/Tools/pfa/iprscan/) [32]. The conserved amino acids were analyzed by protein alignment using such tools as ClustalW [18] and checked manually [33]. Phylogenetic analysis was performed by using ClustalW [18] and MEGA 5.0 [34] for neighbor-joining analysis. The reliability of these tree topologies was evaluated with bootstrapping support of 1000 replicates [34].

### 4.4. RNA Extraction and PCR Analysis

Total RNA was extracted from plant tissues in Trizol reagent (Invitrogen, New York, CA, USA) and pretreated with RNase-Free DNase (Promega, Madison, WI, USA) to eliminate genomic DNA contamination. RNA integrity was analyzed on 1% agarose gel. RNA quantity was determined with a NanoDrop 2000C spectrophotometer (Thermo Scientific, Waltham, MA, USA). Total RNA was reverse-transcribed with Reverse Transcriptase MMLV (D6130; Takara, Dalian, China) from flowers, carpels, bark-free roots, and bark. PCRs were performed in triplicate by using SYBR Premix Ex Taq kits (DRR066A; TaKaRa, Dalian, China) and following the manufacturer’s instructions on an ABI 7500 Real-Time PCR System (ABI, Austen, TX, USA). Gene-specific primers (Table S2) were designed using primer designing tools, particularly Primer3 (http://frodo.wi.mit.edu/primer3/) [35]. Plactin (GenBankJN105299; Available online: http://www.ncbi.nlm.nih.gov) was chosen as an endogenous control in studying gene expressions in various samples of *P. lactiflora*. Standard deviations were calculated from the three PCR replicates. The specificity of amplification was assessed by melting curve analysis, and the relative abundance of genes was determined based on the comparative Ct method as suggested in ABI 7500 Software v2.0.1 (ABI, Austen, TX, USA).

### 4.5. Chemical Analysis

The dried samples were separately comminuted with a miller. Each solid sample (40 mesh, 0.50 g) was accurately weighed and extracted with 50 mL of 50% aqueous methanol by ultrasonication for 30 min. The extract was cooled to 25 °C, diluted with 50 mL 50% aqueous methanol, and then filtered with a 0.45-μm Millipore filter membrane (Millipore, MA, USA).

The HPLC system used was an Agilent 1200 LC Series (Agilent Technologies, Palo Alto, CA, USA) consisting of an online vacuum degasser (G1379B), an SL binary pump (G1312B), an autosampler (GB67C), a thermostatic column compartment (G1316B), and a diode-array detector (G1315C) coupled with an analytical workstation. The column configuration was an Agilent TC-C18 column (5 μm, 250 mm × 4.6 mm). The sample injection volume was 10 μL. The detection wavelength was set at 230 nm for analysis with the flow rate at 1.0 mL min^−1^, and the column temperature was maintained at 25 °C. The mobile phase consisted of deionized water/formic acid (A; 98:2, *v*/*v*) and acetonitrile (B). The elution conditions are shown in Table S3. To determine the linearity of the chromatographic techniques, we constructed calibration plots of eight standards on the basis of peak areas (*y*) using seven concentration solutions (*x*). All plots were linear in the examined ranges, and linear ranges were shown at different concentrations for the standard compounds (μg/mL). The *r* value in Table S4 refers to the correlation coefficient of the equation. All standard compounds, which were purchased from the National Institutes for Food and Drug Control, showed good linearity (*r* > 0.9995) in a relatively wide concentration range.

### 4.6. Correlation Analysis

Correlation analysis conducted with SPSS 13.0 (IBM Corporation, http://www.ibm.com/software/analytics/spss/) was dependent on the contents of active compounds and the transcript levels of paeoniflorin and gallic acid biosynthesis related genes in the roots of *P. lactiflora*.

## 5. Conclusions

We examine 24 genes and their expressions involved in the biosynthesis of paeoniflorin and its derivatives in *Paeonia* medicinal materials. We also associated metabolic pathways involved in processing active medicinal compounds to the expressions of their catalytic enzymes. Our study shows that paeoniflorin and its derived aromatic amino acids are predominant in bark.

## Supplementary Information



## Figures and Tables

**Table 1:** Genes related to the biosynthesis of paeoniflorin and gallic acid in *P. lactiflora.*

Enzyme	Name	Contig No.	Length (bp)
2.5.1.54	PLDHS1	HTIP-2010562	2105*
	PLDHS2	HTIP-2057123	2054
4.2.3.4	PLDHQS	HTIP-2010607	1790
4.2.1.10/	PLaroDE1	HTIP-2007063	1963
1.1.1.25	PLaroDE2	HTIP-2057051	1971
2.3.3.10	PLHMGS1	HTIP-2056545	1615*
	PLHMGS2	HTIP-2056640	1661*
1.1.1.34	PLHMGCR	HTIP-2056491	1591
2.7.1.36	PLMVK	HTIP-2009308	1464
2.7.4.2	PLPMK	HTIP-2056266	1494
4.1.1.33	PLMVD1	HTIP-2044815	316
	PLMVD2	HTIP-2049597	479
2.3.1.9	PLACAT	HTIP-2011414	1399*
	PLDXPS1	HTIP-2003301	2518
2.2.1.7	PLDXPS2	HTIP-2003675	2488*
	PLDXPS3	HTIP-2057315	2338*
1.1.1.267	PLDXR1	HTIP-2000861	423
	PLDXR2	HTIP-2006951	1040
2.7.7.60	PLIspD	HTIP-2008416	1345
2.7.1.148	PLCMK	HTIP-2008205	1609*
4.6.1.12	PLIspF	HTIP-2054028	896*
1.17.7.1	PLHDS	HTIP-2007108	2665
1.17.7.2	PLHDR	HTIP-2056592	1637
5.3.3.2	PLIDI	HTIP-2055407	1185

* full-length cDNA.

**Table 2:** Biosynthesis full-length cDNA of paeoniflorin and gallic acid in *P. lactiflora*.

Name	Length (aa)	Molecular weight (KD)	Isoelectric point	Subcellular Localization	Signal peptide	Domains (# of domains)
DHS1	528	58.7	8.06	Mit.	No	DAHP synthetase, class II(IPR002480)
HMGCS1	453	50.6	6.10	Cytosol	No	Hydroxymethylglutaryl-coenzyme A synthase, active site(IPR000590)
HMGCS2	465	51.3	5.98	Cytosol	No	Hydroxymethylglutaryl-coenzyme A synthase, active site(IPR000590)
ACAT	405	41.4	6.15	Cytosol	No	Thiolase (IPR002155)
DXPS2	714	77.0	6.60	Chl.	No	Deoxyxylulose-5-phosphate synthase (IPR005477)
DXPS3	719	78.9	6.17	Cytosol	No	Deoxyxylulose-5-phosphate synthase (IPR005477)
CMK	403	44.5	6.13	Chl.	No	4-diphosphocytidyl-2*C*-methyl-d-erthritol kinase(IPR004424)
IspF	231	25.0	7.89	Chl.	No	2-*C*-methyl-d-erythritol 2,4-cyclodophosphate synthase (IPR003526)

**Table 3:** Correlation of active compound content and gene transcription level in the *P. lactiflora* root.

Enzyme	Gene	Peony lactone glycosides	Paeoniflorin	Benzoic acid	Benzoyl paeoniflorin	Paeonol
Shikimate biosynthesis						
2.5.1.54	2010562	−0.40	−0.71	−0.36	−0.38	−0.60
	2057123	−0.11	0.70	0.68	0.76	0.48
4.2.3.4	2010607	0.04	−0.51	−0.64	−0.57	−0.40
4.2.1.10/	2057051	−0.05	0.72	0.95	0.94	0.68
1.1.1.25	2007063	0.14	0.71	0.88	0.86	0.80
MVA pathway						
2.3.3.10	2056545	−0.63	0.38	0.88	0.83	0.07
	2056640	−0.02	0.89	0.45	0.50	0.30
1.1.1.34	2056491	0.15	−0.08	−0.12	−0.08	0.15
2.7.1.36	2009308	0.55	−0.20	−0.10	−0.19	0.38
2.7.4.2	2056266	−0.62	0.36	0.88	0.84	0.10
2.3.1.9	2011414	0.17	−0.24	−0.64	−0.55	−0.28
4.1.1.33	2044815	−0.50	0.01	0.70	0.62	0.06
MEP/DXP pathway						
2.2.1.7	2003675	0.12	−0.22	−0.55	−0.47	−0.23
	2003301	−0.19	−0.14	0.07	0.12	−0.06
	2057315	0.19	−0.72	−0.75	−0.76	−0.44
1.1.1.267	2000861	−0.22	−0.64	−0.38	−0.37	−0.49
	2006951	−0.21	−0.57	−0.51	−0.47	−0.54
2.7.7.60	2008416	0.71	−0.37	−0.43	−0.48	0.27
2.7.1.148	2008205	−0.07	−0.49	−0.38	−0.33	−0.29
4.6.1.12	2054028	−0.05	−0.48	−0.42	−0.37	−0.31
1.17.7.1	2007108	−0.03	−0.55	−0.43	−0.39	−0.32
1.17.7.2	2056592	−0.15	−0.48	−0.45	−0.39	−0.42
5.3.3.2	2055407	−0.57	−0.30	0.09	0.11	−0.45
