# Supplementary material for: Functional Diversity of Genes for the Biosynthesis of Paeoniflorin and Its Derivatives in Paeonia
Source: Int J Mol Sci. 2013 Sep 9;14(9):18502–19. doi: 10.3390/ijms140918502 (PMC3794792; doi:10.3390/ijms140918502)
Supplement: Supplementary file 1 [file ijms-14-18502-s001.pdf]

Supplementary Information

Table S1. Transcriptomic database, chemical compounds, and medical uses of *P. lactiflora*, *P. suffruticosa*, *P. unicagranatum*, *R. husradicans* and *Coriarianepalensis*.

Species	Name	Samples	Family	Compounds	Chinese material medicine	Reference
Paeonia lactiflora	HTIP	Flower buds	Paeoniaceae	Paeoniflorin, oxypaeoniflorin, benzoylpaeoniflorin, benzoyloxypaeoniflorin, oxybenzoylpaeoniflorin, albiflorin,lactiflorin, paeoniflorigenone, lactinolid, Paeonilactinone, gallpaeoninorin, albilforin, Paeonivayin, benzoieaeid, stearieacid, oleanolieae, hederagenin, betulinic acid, Gallic acid, *et al.*	Bai Shao	[12]
Paeonia suffruticosa	JI	Whole	Paeoniaceae	Paeonolum, Paeoniflorin, Paeonol, Paeonolide, Apiopaeonoside, Benzoylpaeoniflorin, Oxypaeoniflorin, Oleanolic Acid, Hydroxycoumarin, Gallic acid, *et al.*	Dan Pi	[13]

Table S1. *Cont.*

Species	Name	Samples	Family	Compounds	Chinese material medicine	Reference
Punica granatum	JROW	young leaves	Punicaceae	Gallic acid, Humarain, brevifolin, Pomegranatate, strictinin, Punicafolin, Tellimagrandin, Pedunculagin, Punicalin, 2-*O*-galloylpunicali, granatin, Casuarinin, 5-*O*-galloylpunicacortein, Punigluconin, Punicanolic acid, Friedelin, *et al.*	Shi Liu Pi	[14]
Rhus radicans	YUOM	Leaves stems	Anacardiaceae	Urushiol, catechol, tannic acid, gallic acid, fisetin, mangiferolic acid, anacardic acid, fustin, sulfaretin, myricetin, skikimic acid, glucoside, *et al.*	--	[15]
Coriaria nepalensis	NNGU	leaves	Coriariaceae	Braylin, norbraylin, dihydrocoriamyrtin , coriamyrtin, tutin, coriatin, apotutin, hydroxycoriatin, gallic acid, *et al.*	--	[16]

Table S2. Gene-specific primer sequences for gene identification and expression level assessment of qRT-PCR.

Gene	Forward primer (5'-3')	Reverse primer (5'-3')
HTIP-2056491	TGGGTGTGAAAGGTGCGAG	AATGGCAGACATCAACGACAG
HTIP-2000861	GGCTGGAGGGACTATGACTG	TAAACCAAATGATGGCTGC
HTIP-2007108	ACTGAGGCTGGTGAAGGTGAG	CATCCCGGTGCAGGACA
HTIP-2056592	CTTCCTTGCGACCAAACCCT	ATCATCTTCCTCCGTGGCTGTA
HTIP-2003301	ACTCGTCTACCTTTGCTGCTACT	TCTTTGCCAACTGCTACCCT
HTIP-2003675	AGGGAGTTACCAAACAAATCGG	CGGTCTGGGAAACGGCG
HTIP-2057315	TGGACCCACCATACCACG	AATGACAGCGGACACTCCTTAC
HTIP-2011414	CGGGAGGCAGAGGGAAACC	TTGATTAGCAAGAGCCACGACAG
HTIP-2056545	TGGAAGCGGTCTATCTGCG	CAGGAGGAAACTCGTGTCTTGAT
HTIP-2056640	TGGGTGGAAAGTAACTCGTGG	AAGGTCAGGCTTGTAGAAGTCATAG
HTIP-2010562	TGCCCAGCACCACGGAC	CAGAACAAGCCAGGGAGAATAAC
HTIP-2057123	CGCACGAATGCTTGCTTTTAC	GATGGTAATCCTTCCTGGCTTG

Table S2. *Cont.*

Gene	Forward primer (5'-3')	Reverse primer (5'-3')
HTIP-2009308	TGGATTGTTCGGAAGGAGG	TCGGATGTTAGGCGTGC
HTIP-2008205	TGAGAACAGAGTGAGCCTTGAA	CTCCGTCCATCCTGCCAC
HTIP-2056266	TGAGATGGGCGAGGGTGC	TGAACTGAAGAAATGGCTGATACAAC
HTIP-2008416	ATGGACTTGGTAGGAAATGGGT	AGCAGTTGGCGATGGCAC
HTIP-2044815	GATTCATCCGCCATTTCCAG	CCGTAACAACTCCCGCACTC
HTIP-2049497	GTCCCGCATTCCTGTGGTG	TGCTGGGTTGGCTTCTTCC
HTIP-2007063	AGCGTTCCCTTGGCTACATC	ACCGCCTTCCCACTTCG
HTIP-2057051	AGGGTGGGCAAAGGAGAC	CAAGTACAAGTGGAGTTCGGAG
HTIP-2010607	CCCTCCTCCCACTCTTCCTTT	CTTGAGCCGAACTCGCACAT
HTIP-2054028	TGCTTCATTGCGTTGTGGAT	CAAGTTCATAACCTGCCTCGTC
HTIP-2055407	TTTCACCCAAATCTCACATCG	ACAACATTATCATTCTCGTCCACT
HTIP-2006951	TGGCAACCTCAATGACACC	CTGGTTCAAATGTGACTCTTCTT
actin	GCAGTGTTCCCCAGTATT	TCTTTTCCATGTCATCCC

Table S3. HPLC conditions.

T (min)	A (%)	B (%)
0	95	5
25	93	7
35	88	12
65	86.5	13.5

Note: T, Retention time; A, mobile phase deionized water- formic acid (98:2, *v*/*v*); B, mobile phase acetonitrile.

Table S4. Chemical formulas of active compounds in *Paeonia*.

Compounds	Formula	Linearity correlation (*R*)
Peony lactone glycosides	$y = 2E + 06x - 19052$	0.9995
Paeoniflorin	$y = 1E + 06x + 18652$	0.9995
Benzoic acid	$y = 6E + 06x - 4934$	0.9995
Benzoyl paeoniflorin	$y = 6E + 06x + 41796$	0.9995
Paeonol	$y = 4E + 06x + 15731$	0.9995

Note: Calibration plots of four standards were constructed on the basis of peak areas (*y*) using seven different concentration solutions (*x*). All plots were linear in the examined ranges, and linear ranges had been shown as the concentration of the standard compounds (1 g mL^{-1}). The *R* referred to the correlation coefficient of the equation. The standard compounds were purchased from National institutes for food and drug control, China.

Table S5. Orthologous genes related to the biosynthesis of paeoniflorin and gallic acid in *P. suffruticosa*, *Punicagranatum*, *Rhusradicans,* and *Corarianepalensis.*

Enzyme	***Paeonia suffruticosa***	***Punica granatum***	***Rhus radicans***	***Coriaria nepalensis***
	**Contig No.**	**Contig No.**	**Contig No.**	**Contig No.**
2.5.1.54	JI449682	JROW-2002529	YUOM-2000104	NNGU-2014951
	JI444900		YUOM-2001495	NNGU-2094439
	JI444901		YUOM-2010426	
4.2.3.4	JI457522	JROW-2064854	YUOM-2006774	NNGU-2001397
				NNGU-2001398
				NNGU-2001400
4.2.1.10/1.1.1.25	JI456716	JROW-2001105	YUOM-2000314	NNGU-2094496
	JI448700	JROW-2001106	YUOM-2000315	
	JI454716	JROW-2009754	YUOM-2011625	
		JROW-2002635	YUOM-2038955	
			YUOM-2012212	
2.3.3.10	JI446744	JROW-2009760	YUOM-2011469	NNGU-2023038
	JI448691			NNGU-2023039
				NNGU-2025722
1.1.1.34	JI454933	JROW-2064932	YUOM-2004227	NNGU-2008975
			YUOM-2039378	NNGU-2008976
				NNGU-2008977
2.7.1.36	JI451094	JROW-2006872	YUOM-2004663	NNGU-2001935
				NNGU-2005819
				NNGU-2005820
2.7.4.2	JI455091	JROW-2028708	YUOM-2039067	NNGU-2091117
				NNGU-2092888
4.1.1.33	JI445250	JROW-2003445	YUOM-2038811	NNGU-2094300
		JROW-2052506		
2.3.1.9	JI447640	JROW-2060754	YUOM-2001553	NNGU-2094093
	JI451723		YUOM-2001554	NNGU-2094232
	JI447641		YUOM-2037851	
2.2.1.7	JI447268	JROW-2001269	YUOM-2010080	NNGU-2025412
	JI452657	JROW-2009533	YUOM-2039542	NNGU-2026482
	JI447269			NNGU-2094641
	JI447267			NNGU-2024936
	JI447843			
1.1.1.267	JI447843	JROW-2006718	YUOM-2005209	NNGU-2024936
2.7.7.60	JI449471	-	YUOM-2036678	NNGU-2014133
2.7.1.148	JI456924	JROW-2008008	YUOM-2037480	NNGU-2006912
				NNGU-2006914
4.6.1.12	JI453204	JROW-2064030	YUOM-2037098	NNGU-2000688
				NNGU-2000689
				NNGU-2000690

Table S5. *Cont.*

Enzyme	*Paeonia suffruticosa*	*Punica granatum*	*Rhus radicans*	*Coriaria nepalensis*
	Contig No.	Contig No.	Contig No.	Contig No.
1.17.7.1	JI448239	JROW-2005509	YUOM-2003220	NNGU-2013350
		JROW-2063357	YUOM-2003221	NNGU-2013351
		JROW-2063526		NNGU-2013743
				NNGU-2013744
1.17.7.2	JI447937	JROW-2005797	YUOM-2038387	NNGU-2026367
5.3.3.2	JI448125	JROW-2000471	YUOM-2008052	NNGU-2015100
				NNGU-2017126

Table S6. Correlation of active compounds content in roots of *Paeonia lactiflora.*

Correlation (*R*)	Peony lactone glycosides	Paeoniflorin	Benzoic acid	Benzoyl paeoniflorin	Paeonol
Peony lactone glycosides	1.00				
Paeoniflorin	−0.01	1.00			
Benzoic acid	−0.34	0.72	1.00		
Benzoyl paeoniflorin	−0.34	0.78	0.99	1.00	
Paeonol	0.67	0.55	0.46	0.46	1.00

Figure S1. Compounds in *P. lactiflora* roots.

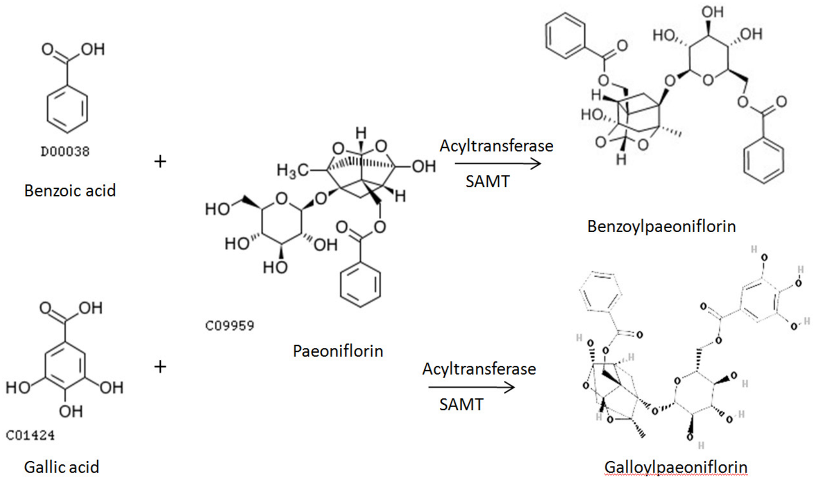

Figure S2. Phylogenetic relationship of plant DXPSs (E2.2.1.7). The rooted neighbor-joining tree was constructed with ClustalW [28] and all of genes were divided into three groups (I, II, and III). HTIP, *P. lactiflora*; JI, *P. suffruticosa*; JROW, *Punica granatum*; YUOM, *Rhus radicans*; NNGU, *Coriaria nepalensis*; AT, *Arabidopsis thaliana*; Os, *Oryza sativa*.

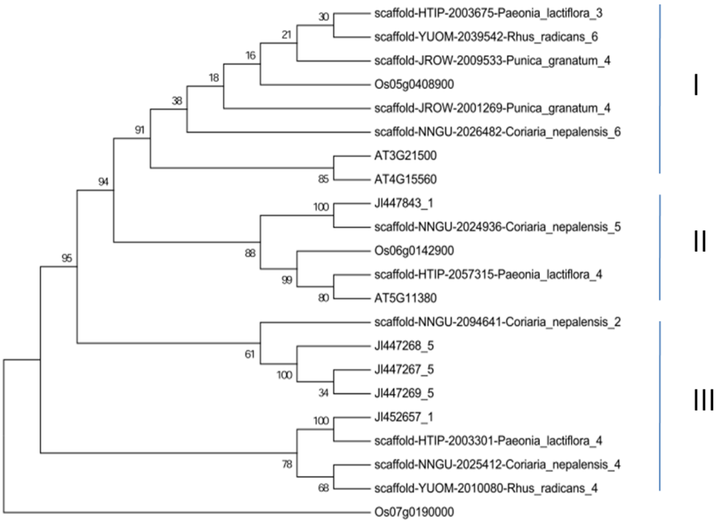

Figure S3. Phylogenetic relationship of plant gene families (A, DXR, E1.1.1.267; B, IspD, E2.7.7.60; C, CMK, E2.7.1.148; D, IspF, E4.6.1.12; E, HDS, E1.17.7.1; F, HDR, E1.17.7.2; and G, IDI, E5.3.3.2). The rooted neighbor-joining tree was constructed with ClustalW. HTIP, *P. lactiflora*; JI, *P. suffruticosa*; JROW, *Punicagranatum*; YUOM, *Rhusradicans*; NNGU, *Coriarianepalensis*; and AT, *Arabidopsis thaliana*.

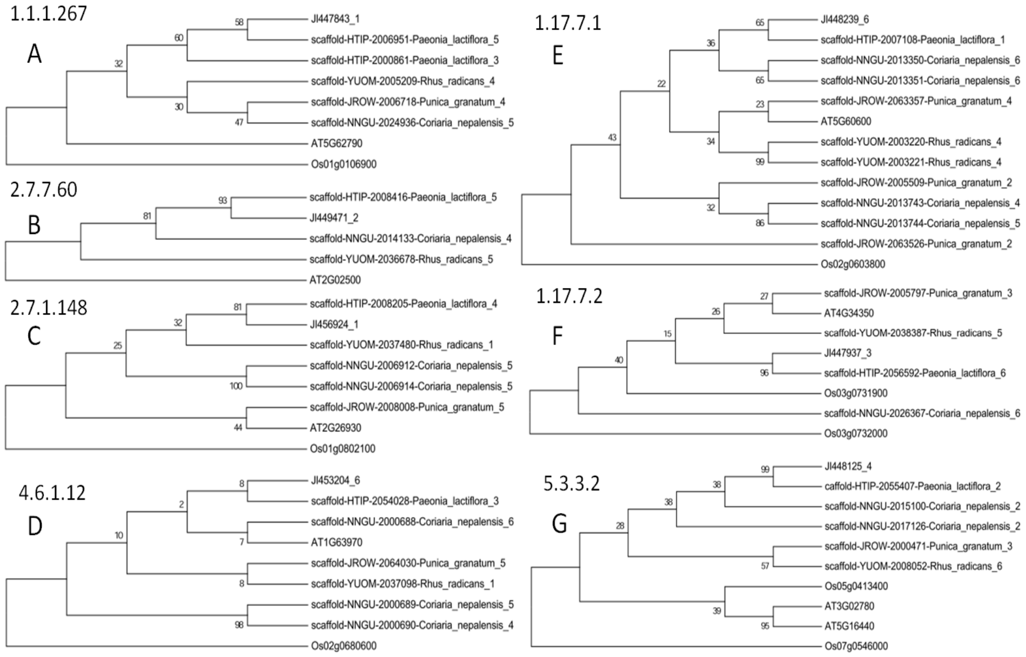

Figure S4. The transcription levels of gene families (A, DXR, E1.1.1.267; B, IspD, E2.7.7.60; C, CMK, E2.7.1.148; D, IspF, E4.6.1.12; E, HDS, E1.17.7.1; F, HDR, E1.17.7.2; and G, IDI, E5.3.3.2) in different tissues and flower of *P. lactiflora*. F, flower; C, carpel; RU, RM, and RL, the upper, middle, and lower portions of the bark-free root; BU, BM, and BL, the upper, middle, and lower portions of the root bark.

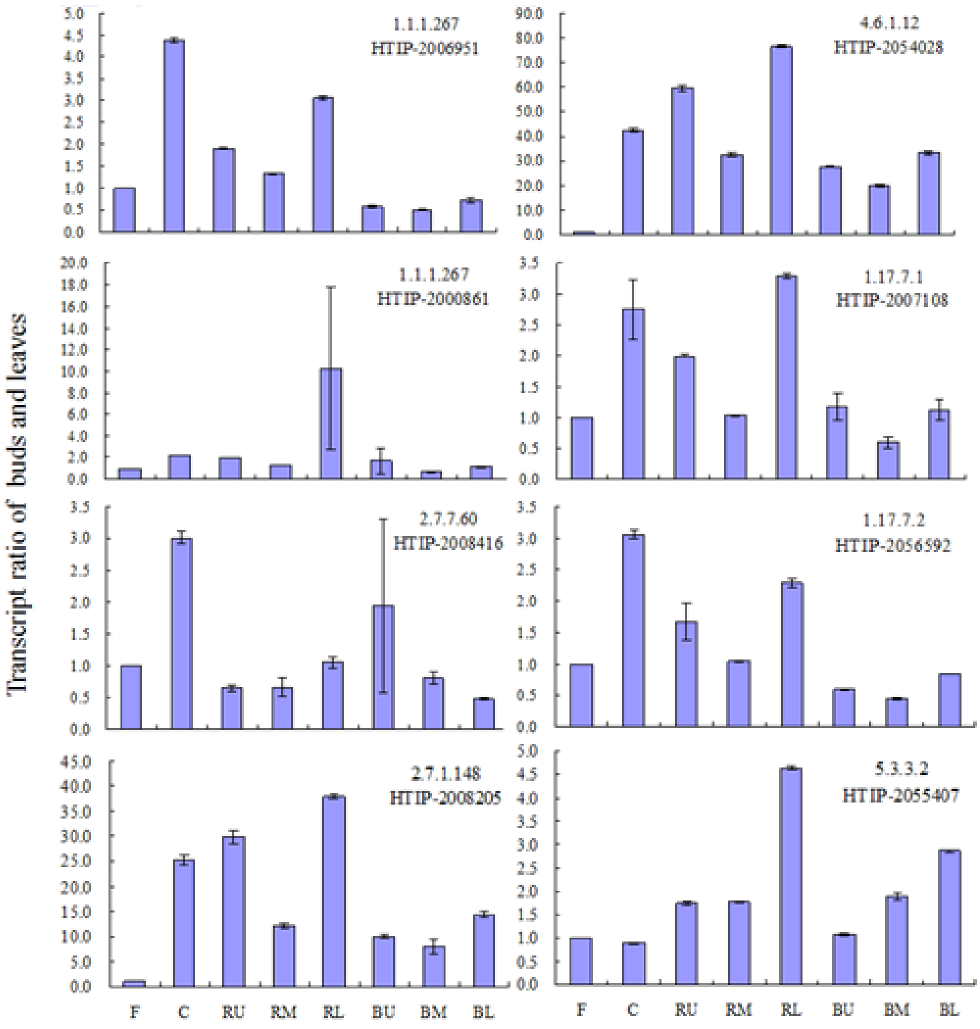

Figure S5. Transcription levels of the DXPS (E2.2.1.7) gene family in different tissues and organs of *P. lactiflora*. F, flower; C, carpel; RU, RM, and RL, the upper, middle, and lower portions of the bark-free root; BU, BM, and BL, the upper, middle, and lower portions of the root bark.

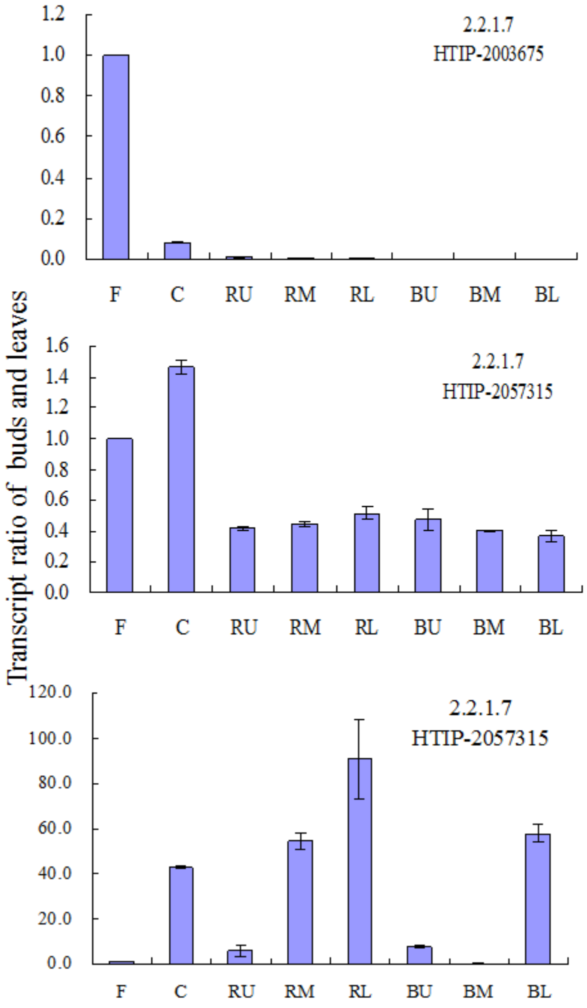

© 2013 by the authors; licensee MDPI, Basel, Switzerland. This article is an open access article distributed under the terms and conditions of the Creative Commons Attribution license (http://creativecommons.org/licenses/by/3.0/).
